# The higher dietary energy-to-fiber ratio is associated with an increased risk of poor blood pressure control among patients with essential hypertension: a cross-sectional study

**DOI:** 10.3389/fnut.2026.1763181

**Published:** 2026-05-05

**Authors:** Jifang Zhu, Yiqing Jiang, Xiaomin Ma, Xiaohua Wang, Minxia Lu

**Affiliations:** 1 Department of Cardiology, The First Affiliated Hospital of Soochow University, Suzhou, China; 2 Department of Nursing, The First People’s Hospital of Kunshan, Suzhou, China

**Keywords:** blood pressure control, dietary fiber, energy intake, essential hypertension, nutrients

## Abstract

**Introduction:**

Dietary fiber (DF) and energy intake are two noted factors in the dietary management of hypertensive patients, which are associated with blood pressure (BP) control. We hypothesized that a higher energy-to-fiber ratio (EFR) is a stronger associated factor for BP control in the hypertensive population.

**Methods:**

Subjects from one comprehensive hospital and one community clinic in China were included in this cross-sectional study (*n* = 454). Dietary intake was measured by 2 non-consecutive 24 h dietary recalls. The EFR was calculated from the ratio of dietary energy (kcal/d) to DF (g/d).

**Results:**

According to the quartile grouping of EFR from low to high, the participants were divided into Q1, Q2, Q3, and Q4 groups, respectively. Poor BP control was defined as systolic BP (SBP) ≥ 140 mmHg and diastolic BP (DBP) ≥ 90 mmHg. In binary logistic regression, taking Q1 as the reference, higher EFRs in Q3 (OR, 2.112; 95% CI, 1.110–4.019) and Q4 (OR, 7.233; 95% CI, 3.709–14.105) were associated with higher rates of poorly controlled SBP and higher EFRs in Q4 (OR, 3.546; 95% CI, 1.836–6.848) were associated with higher rates of poorly controlled DBP, after adjusting for covariates. The correlations between EFR and BP control were stronger than DF or energy alone. A high EFR was a stronger associated factor for poor BP control among patients with essential hypertension.

**Conclusions:**

Given the strong association between diet and hypertension, the indicator EFR should be considered when assessing hypertensive patients’ nutritional status and formulating nutritional strategies.

## Introduction

Hypertension or elevated blood pressure (BP) is a major risk factor for cardiovascular disease and all-cause mortality worldwide due to damage to target organs and subsequent serious complications such as stroke, acute myocardial infarction, and heart failure (1, 2). Poor BP control in hypertensive patients is a growing public health problem in China. Among 245 million hypertensive patients in China reported in 2020, the BP control rate remained very low, only about 15.3% (3). Considering the disease burden and high prevalence of hypertension, it is necessary to find out the additional modifiable risk factors for poor BP control that could be beneficial in developing potential preventative measures.

In China, suboptimal diet is an important preventable risk factor for mortality (4) and non-communicable diseases including hypertension (5). Dietary fiber (DF) refers to polysaccharides originating from plant-based foods that are not digested by the endogenous enzymes in the small intestine of humans (6). DF can reduce inflammation/oxidative stress and improves insulin resistance (7, 8), which improve vascular endothelial function (9, 10), resulting in a decrease in BP (11). DF deficiency is a risk factor that contributes to higher BP (12). DF supplementation effectively relieved the 24-h ambulatory BP (13). Thus, the importance of increasing DF-rich foods intake such as fruits, vegetables, and whole grains has been emphasized in the Dietary Approaches to Stop Hypertension (DASH) diet and the Mediterranean diet, two of the most frequently recommended dietary strategies for patients with hypertension (14).

Total energy intake, another noted factor of dietary pattern, may impact BP control (15). In recent years, consumption of high-energy-density foods such as animal fats, sugar-sweetened beverages, and processed meats has only increased in China (16, 17). Excessive energy consumption can lead to over accumulation of fat cells and increased inflammation, oxidative stress, and insulin resistance (18–20), which cause a decrease in the bioavailability of nitric oxide (NO) (21, 22). NO is an important vasodilator released by the vascular endothelium (23). Thus excessive energy consumption leads to vascular endothelial dysfunction (24), which is detrimental to BP control in hypertensive patients (25). However, Dong et al. did not find a correlation between energy intake and BP control in patients with essential hypertension (26).

Dietary fiber (DF) may interact with other macronutrients in the intestine, thereby modulating their metabolic and pro-inflammatory effects, which in turn influence blood pressure control (8, 27), which can affect BP control. Different diets and ratios of dietary components and different levels of nutrients and their ratios may be an important consideration in chronic disease (28). Since excessive energy and less DF intake partly share the pathogenesis of hypertension, DF supplementation may contribute synergistically with energy restriction to good BP control in the essential hypertensive population. Several previous studies have found that energy restriction combined with DF supplementation was more effective in lowering BP than energy restriction alone among non-hypertensive participants (29–31). However, the association between dietary energy-to-fiber ratio (EFR) and BP control remains unknown, and we hypothesized that a higher dietary EFR is a stronger risk factor for BP control in the hypertensive population.

Although several dietary quality indices, such as the Dietary Approaches to Stop Hypertension (DASH) score and the Mediterranean diet score, have demonstrated beneficial effects on blood pressure control, their application in clinical and epidemiological settings often requires comprehensive dietary assessments, complex scoring algorithms, and detailed nutrient profiling, which may limit their feasibility in routine practice. In contrast, ratio-based indicators derived from key dietary components have emerged as simpler alternatives that can capture the balance between detrimental and protective nutritional factors. The EFR integrates two biologically relevant and functionally opposing dietary components: total energy intake, which is associated with adiposity, inflammation, and metabolic dysregulation, and dietary fiber intake, which exerts anti-inflammatory, metabolic, and vascular protective effects. By combining these two dimensions into a single metric, EFR may better reflect the overall dietary pattern and its net physiological impact than either component alone. Importantly, while energy intake alone showed inconsistent associations with blood pressure in previous studies, and dietary fiber alone does not account for excessive caloric exposure, EFR provides a composite measure that captures their interaction and relative balance. Moreover, compared with other nutrient ratios, EFR may have broader applicability because total energy intake reflects multiple macronutrient sources and dietary behaviors, including consumption of high-energy-density foods. Therefore, EFR may serve as a practical, quantitative, and clinically accessible indicator for evaluating dietary quality and identifying individuals at higher risk of poor blood pressure control. Therefore, in the current study, we conducted a cross-sectional study among patients with hypertension to comprehensively investigate the association between dietary EFR and blood pressure control, aiming to assess its potential as a simple and clinically applicable indicator of dietary quality, while rigorously adjusting for relevant demographic, clinical, and nutritional confounders.

## Materials and methods

### Study design and population

This is a population-based cross-sectional study carried out from June 2019 to October 2020. The patients with hypertension who met the inclusion and exclusion criteria were recruited for investigation through convenient sampling in the First Affiliated Hospital of Soochow University and Jinchang Community, China. PASS 15.0 software for single population proportion sample size prediction was used with bilateral α = 0.05, 5% margin of error, 46.7% poor BP control rate in Suzhou in Qi et al.’s study, and 10% for possible non-response was taken to determine a final sample size of 445. This study was conducted in accordance with the declaration of Helsinki and with approval from the Ethics Committee of the First Affiliated Hospital of Soochow University (No. ECSU-2019000148). All participants provided written informed consent.

The study recruited participants who met the following criteria: (1) diagnosed with essential hypertension according to the 2018 Chinese guidelines for the management of hypertension (32); (2) age ≥ 18 years old; (3) having sufficient communication ability to complete questionnaires independently or with assistance; (4) providing informed consent and volunteered to participate in this study. The exclusion criteria were listed as follows: (1) secondary hypertension; (2) with serious physical comorbidities or complications (e.g., malignant tumors, severe heart, kidney, and liver failure); (3) women who were pregnant and lactating; (4) cognitive impairment; (5) participated in other research.

### Blood pressure measurement

After participants had rested for at least 5 min, their BP was measured using an electronic monitor (Omron HEM-8102A, Omron Corporation, Shanghai, Japan) on the arm. Participants were asked to keep seated in the community service room or hospital demonstration classroom. A repeat office BP measurement was performed after a 5-min interval. The average of two measurements was the final BP level. Poor BP control was defined as systolic blood pressure (SBP) ≥ 140 mmHg and/or diastolic blood pressure (DBP) ≥ 90 mmHg (33).

### Dietary nutrient measurement

The information of energy, dietary fiber, and other nutrient intake were obtained through two 24-h dietary recalls (a day on a weekend and a day on a weekday) (34). The 24-h dietary recall is a method that reviews and describes the consumption of all foods during the day before the moment of investigation. Participants were asked to recall all foods and beverages consumed within the previous 24 h, including staple foods (e.g., rice, wheat products), vegetables, fruits, legumes, meat and poultry, fish, dairy products, snacks, and beverages. Dietary fiber intake was primarily derived from plant-based foods such as whole grains, vegetables, fruits, and legumes, in accordance with the Chinese Food Composition Tables. Energy intake was calculated using standardized nutrient conversion coefficients embedded in the Beijing Feihua Nutrition Software (V2.7.6.10), which integrates food composition data to estimate total daily caloric intake. The energy-to-fiber ratio (EFR) was calculated as total daily energy intake (kcal/d) divided by dietary fiber intake (g/d). Under the guidance of two trained researchers, with reference to the standard food portion reference chart from the Dietary Guidelines for Chinese Resident (the 2016 edition) for standard food portions to collect the first 24-h dietary data face-to-face. The second 24-h dietary recall was collected via telephone or WeChat (a widely used mobile messaging application in China) 2–3 days later (35). We took the average of nutrient intake during two recording days using Beijing Feihua Nutrition Software (V2.7.6.10, Beijing, China) as the final values. EFR was defined as the ratio of dietary energy (kcal/d) to fiber (g/d) intake. According to the quartile grouping of EFR from low to high, the participants were divided into Q1, Q2, Q3, and Q4 groups, respectively.

### Assessment of demographic and clinical data

Demographic and clinical data were obtained through general questionnaires, which were developed by our team, including demographic indicators (i.e., age, sex, marital status, occupational status, education degree, medical payment method, regular exercise, duration and quality of sleep, smoking status, alcohol drinking status) and clinical indicators [i.e., body mass index (BMI), constipation, duration of hypertension, taking antihypertensive drugs, complication, and comorbidity]. Exercise was defined as: exercising three or more times a week for ≥20 min each time and for more than three consecutive months (36). Sleep quality was a subjective perception of the patient and was self-assessed by the patient based on a Visual Analog Scale. The score of 0–3, 4–6, and more than 7 were categorized as poor, fair and good sleep quality. BMI was calculated by dividing the weight (kg) by the square of the height (m^2^). Complications of hypertension were the occurrence of another disease or condition caused by increased BP during the development of hypertension among participants, including stroke, cerebral hemorrhage, myocardial infarction, frequent angina, aortic coarctation, renal insufficiency, and so on, which were listed in the questionnaire (37). Complications referred to the co-existence of one or more diseases or clinical conditions unrelated to BP (38).

### Data collection process

First, the researcher explained the purpose, significance and methods of the study to participants who met the inclusion criteria, and instructed them to sign the informed consent form. Second, the height and weight of the patients were measured and the patients filled out the general questionnaire under the uniform instruction words of the researcher. Third, the researcher measured the patient’s office BP two times with a 5 min interval. Fourth, the researcher collected the patient’s 24-h dietary data of previous day and obtained their contact ways (telephone/weChat). After 2–3 days of the survey, the investigator obtained the patient’s second 24-h dietary data via telephone interview/WeChat.

### Statistical analyses

1. Participants’ characteristics were described as mean ± standard deviation (SD) or median (25th and 75th percentiles) for continuous variables and frequency (percentages) for categorical variables.
2. Characteristics of participants on BP were compared using *t* test for continuous variables if normally distributed, otherwise, the Mann-Whitney U test was used. Pearson’s Chi-square test or Fisher’s exact test for categorical variables.
3. To compare the rate of poor controlled BP among the Q1–Q4 groups, Pearson’s Chi-square test was used.
4. To initially explore trends between EFRs and BP control and whether there is a potential nonlinear relationship, we used restricted cubic splines (RCS) with 4 knots.
5. Multivariate binary logistic regression models were fitted to explore the associations between EFRs and the rates of the poor controlled BP, adjusting for control covariates (the indicators with *p* < 0.10 in the demographic, clinical and nutritional data). Three stratified covariates were adjusting for SBP and DBP, respectively: Model 1 adjusted for demographic indicators; model 2 adjusted for covariates of model 1 + clinical indicators + nutrient indicators. Key lifestyle and socioeconomic confounders, including smoking status, alcohol consumption, regular exercise, and education level, were incorporated into the multivariable models based on their clinical relevance and statistical significance. A directed acyclic graph (DAG) was constructed to guide the selection of covariates and to minimize potential confounding bias (Supplementary Figure 1).

Statistical significance was defined as *P* < 0.05. RCS statistical analyses were conducted using the R software (version 4.2.1^1^), and other analyses were performed using SPSS 25.0 software (SPSS, Inc., Chicago, IL, United States).

## Results

### Demographic and clinical characteristics

**FIGURE 1:**
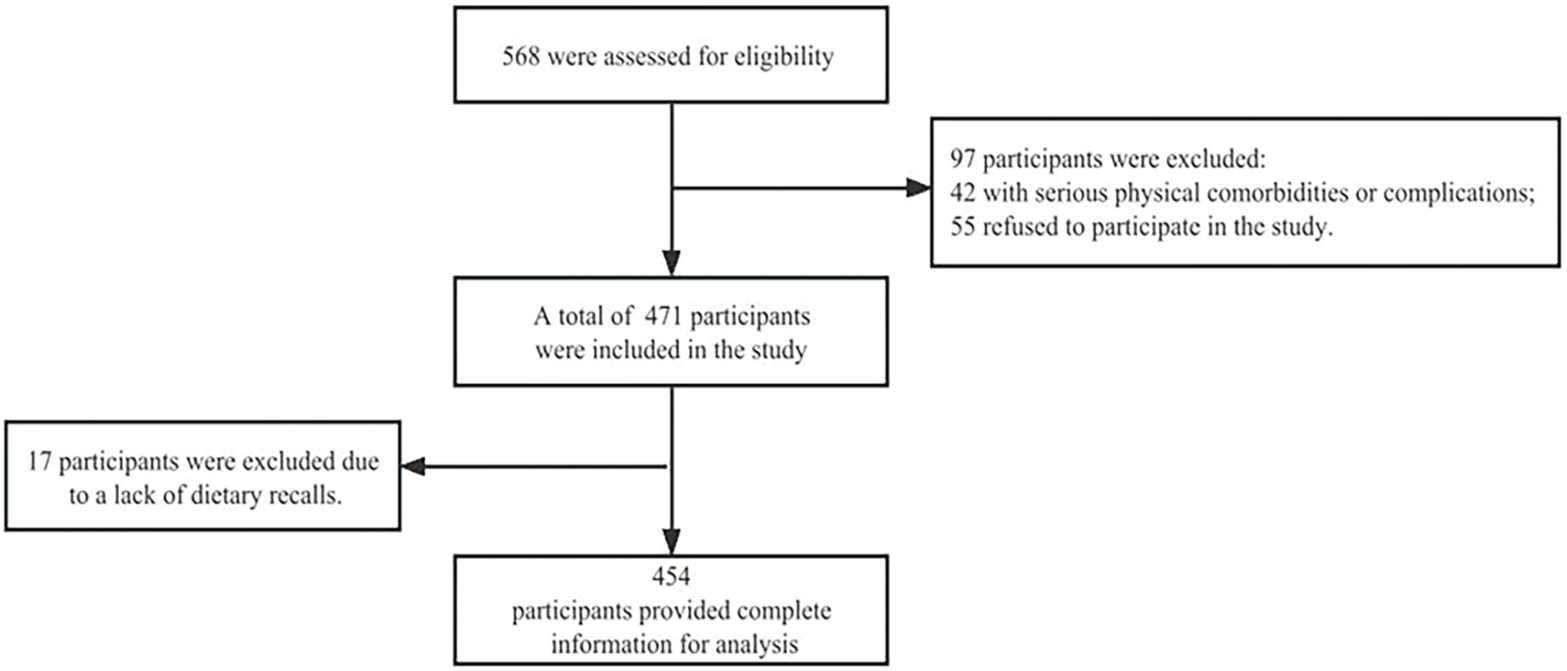
Flowchart of participant selection for this study.

In this study, we initially recruited 568 participants to be assessed for eligibility. Of these, 97 were excluded according to the criteria described in Figure 1. Further, 17 participants who lacked 2–24 h dietary recalls were excluded. Finally, 454 (51.3 ± 12.7 years) participants were included in the final analysis, 285 males (62.8%) and 169 females (37.2%). The participant selection process is shown in Figure 1. The mean BMI was 25.4 ± 4.8, and 299(65.8%) participants were overweight. The socio-demographic, clinical data, and the characteristics of consumed nutrients of the good and poorly controlled SBP and DBP populations are shown in Table 1. The poor SBP and DBP controlled rates were 40.5% and 39.0%, respectively. Poor SBP control was more common among participants who were married, constipated, had a short duration of hypertension, did not take medication, had less carbohydrate and DF intake, and had more sodium intake. The poorly controlled DBP was more common among those who were younger, male, on the job, had a high school or higher education, smoked, drank alcohol; did not take medication, had a short duration of hypertension, and had less DF intake.

**TABLE 1:** The socio-demographic, clinical characteristics and nutrients of hypertensive participants (*n* = 454).

Characteristics		SBP x ± S/M (P_25_, P_75_)/*n* (%)				DBP−x ± S/M (P_25_, P_75_)/*n* (%)			
		Good control (*N* = 270)	Poor control (*N* = 184)	*t/χ2 /z*	*P*	Good control (*N* = 277)	Poor control (*N* = 177)	*t/χ2 /z*	*P*
Socio-demographic data									
Age (y)		52.1 ± 11.6	50.1 ± 14.0	1.619^a^	0.11	54.6 ± 12.2	46.1 ± 11.6	7.401^a^	<0.001^***^
Sex	Male	172 (63.7)	113 (61.4)	0.246^b^	0.62	163 (58.8)	122 (68.9)	4.697^b^	0.030*
	Female	98 (36.3)	71 (38.6)	–	–	114 (41.2)	55 (31.1)	–	–
Marital status	Single	17 (6.3)	1 (0.5)	11.209^d^	0.001^**^	13 (4.7)	5 (2.8)	1.219^d^	0.62
	Married	250 (92.6)	182 (98.9)	–	–	261 (94.2)	171 (96.6)	–	–
	Others	3 (1.1)	1 (0.5)	–	–	3 (1.1)	1 (0.6)	–	–
Occupational status	On the job	164 (60.7)	119 (64.7)	1.889^d^	0.39	141 (50.9)	142 (80.2)	41.394^d^	<0.001^***^
	Retired	103 (38.1)	61 (33.2)	–	–	131 (47.3)	33 (18.6)	–	–
Awaiting employment	3 (1.1)	4 (2.2)	–	–	5 (1.8)	2 (1.1)	–	–
Education degree	Below high school	130 (48.1)	77 (41.8)	1.751^b^	0.19	150 (54.2)	57 (32.2)	20.972^b^	<0.001^***^
	High school and above	140 (51.9)	107 (58.2)	–	–	127 (45.8)	120 (67.8)	–	–
Medical insurance	No	11 (4.1)	12 (6.5)	1.363^b^	0.24	12 (4.3)	11 (6.2)	0.796^b^	0.37
	Yes	259 (95.9)	172 (93.5)	–	–	265 (95.7)	166 (93.8)	–	–
Regular exercise	Yes	69 (25.6)	50 (27.2)	0.148^b^	0.70	71 (25.6)	48 (27.1)	0.123^b^	0.73
	No	201 (74.4)	134 (72.8)	–	–	206 (74.4)	129 (72.9)	–	–
Duration of sleep (h)	–	–	6.8 ± 1.1	0.342^a^	0.73	6.8 ± 1.2	6.9 ± 1.1	−1.072^a^	0.29
Quality of sleep	Good/fair	249 (92.2)	168 (91.3)	0.123^b^	0.73	254 (91.7)	163 (92.1)	0.022^b^	0.88
	Poor	21 (7.8)	16 (8.7)	–	–	23 (8.3)	14 (7.9)	–	–
Smoking status	Yes	68 (25.2)	49 (26.6)	0.119^b^	0.73	57 (20.6)	60 (33.9)	10.017^b^	0.002^**^
	No	202 (74.8)	135 (73.4)	–	–	220 (79.4)	117 (66.1)	–	–
Alcohol drinking	Yes	63 (23.3)	46 (25.0)	0.167^b^	0.68	51 (18.4)	58 (32.8)	12.201^b^	<0.001^***^
	No	207 (76.7)	138 (75.0)	–	–	226 (81.6)	119 (67.2)	–	–
Clinical data									
BMI (kg/m^2^)	–	25.3 ± 5.3	25.6 ± 3.8	−0.545^a^	0.59	25.1 ± 5.2	26.0 ± 4.0	−1.921^a^	0.06
Constipation	Yes	18 (6.7)	24 (13.0)	5.300^b^	0.021*	21 (7.6)	21 (11.9)	2.360^b^	0.12
	No	252 (93.3)	160 (87.0)	–	–	256 (92.4)	156 (88.1)	–	–
Duration of HTN (y)	–	5.3 (1.0, 10.0)	2.0 (0.5, 7.0)	−3.688^e^	<0.001^***^	5.0 (1.0, 10.0)	2.0 (0.5, 7.0)	−3.545^e^	<0.001^***^
Taking drugs	Yes	223 (82.6)	128 (69.6)	10.588^b^	0.001^**^	234 (84.5)	117 (66.1)	20.788^b^	<0.001^***^
	No	47 (17.4)	56 (30.4)	–	–	43 (15.5)	60 (33.9)	–	–
Complication	Yes	1 (0.4)	4 (2.2)	3.297^c^	0.07	4 (1.4)	1 (0.6)	0.841^c^	0.36
	No	269 (99.6)	180 (97.8)	–	–	273 (98.6)	176 (99.4)	–	–
Comorbidity	Yes	35 (13.0)	27 (14.7)	0.272^b^	0.60	41 (14.8)	21 (11.9)	0.790^c^	0.37
	No	235 (87.0)	157 (85.3)	–	–	236 (85.2)	156 (88.1)	–	–
Nutrients intake									
Energy (kcal/d)	–	2021.88 ± 395.4	1963 ± 326.6	1.719^a^	0.09	1995.59 ± 384.823	2002.21 ± 346.0	−0.186^a^	0.85
DF (g/d)	–	12.4 ± 5.1	9.6 ± 4.0	6.602^a^	<0.001^***^	11.6 ± 5.0	10.7 ± 4.8	2.009^a^	0.045*
Carbohydrate (g/d)	–	297.4 ± 80.6	270.9 ± 73.1	3.568^a^	<0.001^***^	290.6 ± 77.5	280.6 ± 80.4	1.325^a^	0.19
Protein (g/d)	–	75.5 ± 22.4	71.8 ± 21.6	1.783^a^	0.08	72.6 ± 22.0	76.3 ± 22.3	−1.721^a^	0.09
Fat (g/d)	–	68.6 ± 20.6	68.9 ± 19.1	−0.157^a^	0.88	67.6 ± 20.4	70.4 ± 19.2	−1.438^a^	0.15
Cholesterol (mg/d)	–	337.1 ± 208.1	374.4 ± 225.6	−1.811^a^	0.07	336.4 ± 199.7	377.0 ± 237.5	−1.888^a^	0.06
Sodium (mg/d)	–	1962.6 ± 929.5	2110.0 ± 604.9	-2.046^a^	0.041*	2043.5 ± 814.1	1989.2 ± 820.5	0.691^a^	0.49
Potassium (mg/d)	–	1720.1 ± 419.4	1651.9 ± 485.3	1.552^a^	0.12	1675.5 ± 449.1	1719.0 ± 446.2	−1.009^a^	0.31

Good control was defined as systolic blood pressure (SBP) < 140 mmHg and diastolic blood pressure (DBP) < 90 mmHg; poor control was defined as SBP ≥ 140 mmHg and/or DBP ≥ 90 mmHg.

^***^p < 0.001;

^**^p < 0.01;

*p < 0.05. SBP, systolic blood pressure; DBP, diastolic blood pressure; BMI, body mass index; HTN: hypertension; Taking drugs means taking antihypertensive drugs; DF, dietary fiber.

^a^Independent-samples *T*-test.

^b^Pearson Chi-square.

^c^Yates’ correction Chi-square.

^d^Fisher’s exact test.

^e^Mann–Whitney U; M (P_25_, P_75_), median (25th and 75th percentiles).

### Statuses of energy, dietary fiber intake and EFR

Table 2 shows the statuses of energy, DF intake and EFR of the Q1–Q4 groups. The average daily intakes of energy and DF of total participants were 1998.2 kcal/d and 11.3 g/d, respectively. The average values of energy intake for groups Q1, Q2, Q3, and Q4 were 1989.1 ± 430.6, 1994.3 ± 297.9, 2058.0 ± 393.1, and 1951.6 ± 341.6 kcal/d, respectively, which was no statistically significant difference among the four groups (*P* = 0.20). According to the recommended energy by Dietary Guidelines for Chinese Residents (39), about 83 (18.2%) of participants had excessive energy intake, and most participants’ energy intake was within the recommended ranges. The amount of DF intake (g/d) in the four groups were 17.2 ± 4.9, 11.8 ± 3.2, 9.7 ± 3.9, and 6.3 ± 3.4 g/d, respectively. The DF intake from Q1 to Q4 gradually decreased and the difference among groups was significant (*P* < 0.001).

**TABLE 2:** The status of energy (kcal/d), DF intake (g/d) and EFR (kcal/g) by quartile grouping of EFR among hypertensive participants.

Groups (EFR ranges)	Energy				DF			
	Range	Amount	*F* ^a^	*P*	Range	Amount	*F* ^a^	*P*
Q1 (<149.5)	(1039.0, 3261.0)	1989.1 ± 430.6	1.577	0.20	(7.4, 28.9)	17.2 ± 4.9	265.671	<0.001^***^
Q2 (149.5–< 189.0)	(1042.0, 2814.0)	1994.3 ± 297.9			(5.6, 15.8)	11.8 ± 3.2		
Q3 (189.0–< 246.4)	(1258.0, 3560.0)	2058.0 ± 393.1			(5.9, 18.5)	9.7 ± 3.9		
Q4 (≥246.4)	(1223.0, 2750.0)	1951.6 ± 341.6			(2.7, 9.6)	6.3 ± 3.4		

Q, quintile; Q1, P_0_−<P_25_; Q2, P_25_−<P_50_; Q3, P_50_−P_75_; Q4, P_75_−P_100_.

^***^p < 0.001. DF, dietary fiber; EFR, energy to fiber ratio.

*^a^*Analysis of variance.

For EFR, the average value was 189.0, and 36 participants (7.9%) met the European Heart Network’s EFR recommendation (108 kcal/g) (40). The EFRs of good and poor SBP-controlled groups were 175.7 and 214.3, respectively (*t* = −6.024, *P* < 0.001). The EFRs of good and poor DBP-controlled groups were 180.8, and 201.8, respectively (*t* = −2.714, *P* = 0.007).

### Comparison of the rates of the poorly controlled BP in hypertensive patients with different EFRs

The rates of poorly controlled SBP and DBP in the Q1–Q4 groups are presented in Table 3. From Q1 to Q4, the rates of poorly controlled SBP and DBP increased. Q1 had the least poorly controlled rate (23.9% for SBP and 32.7% for DBP), while Q4 had the highest poorly controlled rates (65.8% for SBP and 49.1% for DBP). The differences in the rates of poorly controlled SBP and DBP among the four groups were statistically significant (SBP: χ^2^ = 46.944, *P* < 0.001; DBP: χ^2^ = 7.902, *P* = 0.048).

**TABLE 3:** The rates of the poor control of blood pressure by quartile grouping of EFR among hypertensive participants.

Group	SBP			DBP		
	Poor control, *N* (%)	*χ^2^*	*P*	Poor control, *N* (%)	*χ^2^*	*P*
Q1	27 (23.9)	46.944	<0.001^***^	37 (32.7)	7.902	0.048^*^
Q2	36 (31.6)			39 (34.2)		
Q3	46 (40.7)			45 (39.8)		
Q4	75 (65.8)			56 (49.1)		

Q, Quintile; Q1, P_0_–<P_25_; Q2, P_25_–<P_50_; Q3, P_50_–P_75_; Q4, P_75_–P_100_.

^***^p < 0.001;

*p < 0.05. EFR, energy to fiber ratio; SBP, systolic blood pressure; DBP, diastolic blood pressure.

### Associations between EFR and control of SBP

**FIGURE 2:**
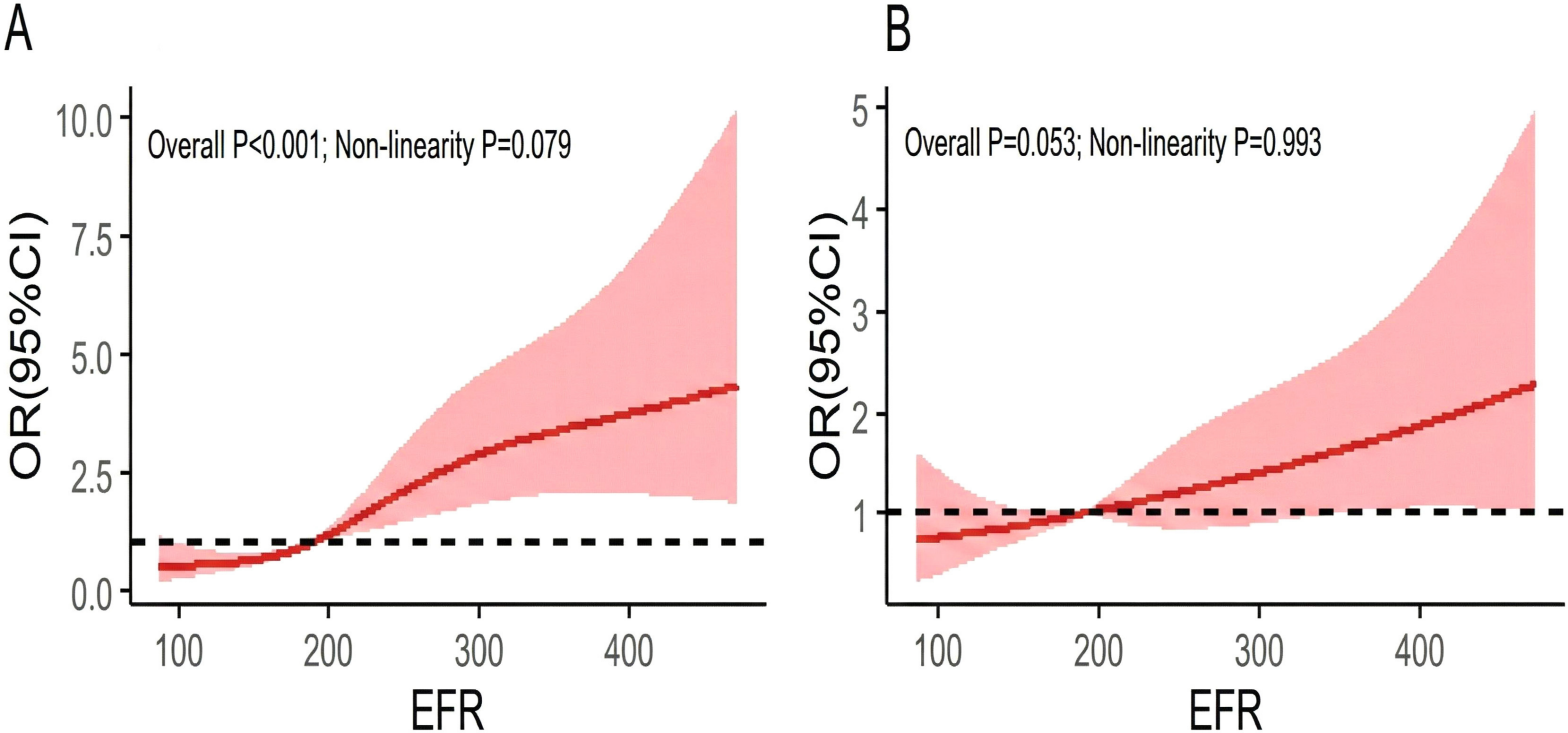
Restricted cubic spline plots of the relationship of EFR and poor BP control. **(A)** for SBP, **(B)** for DBP. The x-axis was EFR and the y-axis was OR (95% CI). The red solid line indicates the estimated poor SBP control risk, and the red shaded area indicates 95% CI. The dashed line at 1.0 indicates the reference. EFR, energy to fiber ratio; BP, blood pressure; SBP, systolic blood pressure; DBP, diastolic blood pressure; OR, odds ratios; CI, confidence intervals.

Restricted cubic splines (RCS) analysis without adjustment for covariates showed a linear relationship between the continuous variable of EFRs and the rates of poorly controlled SBP. Participants in both the third and fourth quartiles of EFRs conferred a higher risk of the rate of poorly controlled SBP, compared with those in the first and second quartiles of EFRs. See Figure 2A.

Table 4 shows the results of binary logistic regression which assessed the odds ratios (OR) with 95% confidence intervals (CI) of the rates of poorly controlled SBP associated with EFRs, adjusted for different potential confounders. Overall, the continuous variable of EFR was positively associated with the rates of poorly controlled SBP after adjusting for potential covariates (OR, 1.008; 95% CI, 1.005–1.011). Taking Q1 as the reference, higher EFRs in Q3 (OR, 2.112; 95% CI, 1.110–4.019) and Q4 (OR, 7.233; 95% CI, 3.709–14.105) were associated with higher rates of poorly controlled SBP, after adjusting for demographics, clinical indicators, and nutrients. The associations of energy and DF intake alone with the poor SBP controlled rate were also explored separately. While the energy alone was not associated with the rates of poorly controlled SBP. For DF, the lower DF intake in Q1 (OR, 5.279; 95% CI, 2.523–11.049) and Q2 (OR, 3.674; 95% CI, 1.845, 7.316) were associated with higher rates of poorly controlled SBP, compared to group Q4.

**TABLE 4:** Odds ratios and 95% confidence intervals for poor SBP control according to the quartile of EFR among hypertensive participants.

Variables		Crude		Model 1		Model 2	
		OR (95% CI)	*P*	OR (95% CI)	*P*	OR (95% CI)	*P*
EFR (kcal/g)	–	1.007 (1.005, 1.010)	<0.001^***^	1.007 (1.005, 1.010)^***^	<0.001^***^	1.008 (1.005, 1.011)^***^	<0.001^***^
Group by quartile of EFR	Q1	1.00 (ref.)	–	1.00 (ref.)	–	1.00 (ref.)	–
	Q2	1.470 (0.819, 2.640)	0.20	1.295 (0.701, 2.394)	0.41	1.418 (0.744, 2.704)	0.29
	Q3	2.187 (1.234, 3.877)	0.007^**^	2.058 (1.121, 3.776)	0.020*	2.112 (1.110, 4.019)	0.023*
	Q4	6.125 (3.429, 10.943)	<0.001^***^	6.201 (3.314, 11.605)	<0.001^***^	7.233 (3.709, 14.105)	<0.001^***^
Group by quartile of energy (kcal/d)	Q1	1.00 (ref.)	–	1.00 (ref.)	–	1.00 (ref.)	–
	Q2	1.215 (0.719, 2.051)	0.47	1.206 (0.710, 2.048)	0.49	1.578 (0.835, 2.984)	0.16
	Q3	0.897 (0.529, 1.521)	0.69	0.908 (0.533, 1.547)	0.72	1.512 (0.723, 3.161)	0.27
	Q4	0.697 (0.406, 1.194)	0.19	0.854 (0.486, 1.501)	0.58	1.744 (0.633, 4.805)	0.28
Group by quartile of DF (g/d)	Q4	1.00 (ref.)	–	1.00 (ref.)	–	1.00 (Ref.)	–
	Q1	4.815 (2.645, 8.765)	<0.001^***^	4.418 (2.337, 8.352)	<0.001^***^	5.279 (2.523, 11.049)	<0.001^***^
	Q2	4.071 (2.226, 7.447)	<0.001^***^	3.521 (1.864, 6.653)	<0.001^***^	3.674 (1.845, 7.316)	<0.001^***^
	Q3	2.158 (1.172. 3.974)	0.014*	1.829 (0.963, 3.475)	0.07	1.819 (0.926, 3.575)	0.08
Covariates	Age (y)	–	–	0.976 (0.959, 0.992)	0.004^**^	–	–
	Duration of HTN (y)	–	–	–	–	0.964 (0.933, 0.996)	0.030*
	Antihypertensive drugs	–	–	–	–	0.434 (0.250, 0.754)	0.003^**^

SBP, systolic blood pressure; EFR, energy to fiber ratio; OR, odds ratios; CI, confidence intervals; Q, quintile; DF, dietary fiber; HTN, hypertension.

^***^p < 0.001;

^**^p < 0.01;

*p < 0.05. Crude: did not adjust anything. Model 1: adjusted for age, sex, marital status. Model 2: adjusted for variables in model 1 + constipation, duration of HTN, taking antihypertensive drugs, complication, cholesterol intake, carbohydrate intake, protein intake, sodium intake.

### Associations between EFR and control of DBP

Same as SBP, RCS analysis without adjustment for covariates also showed a linear relationship between the continuous variable of EFR and the rate of poorly controlled DBP. Participants in both the third and fourth quartiles of EFRs conferred a greater risks of the rate of poorly controlled DBP compared with those in the first and second quartiles of EFRs. See Figure 2B.

Table 5 shows the results of binary logistic regression which assessed the OR with 95% CI of the rates of poorly controlled DBP associated with EFRs, adjusted for different potential confounders. Similarly, in terms of DBP, the continuous variable of EFR was positively associated with the rates of poorly controlled DBP after adjusting for potential covariates (OR, 1.005; 95% CI, 1.002–1.008). Taking Q1 as the reference, higher EFR in Q4 was associated with higher rates of poorly controlled DBP (OR, 3.546; 95% CI, 1.836–6.848), after adjusting for demographics, clinical indicators, and nutrients. While the energy alone were not associated with the rates of poorly controlled DBP. For DF, the lower DF intake in Q1 (OR, 2.575; 95% CI, 1.250–5.305) and Q2 (OR, 2.477; 95% CI, 1.269, 4.837) were associated with higher rates of poorly controlled DBP, compared to group Q4.

**TABLE 5:** Odds ratios and 95% confidence intervals for poor DBP control according to the quartile of EFR among hypertensive participants.

Variables		Crude		Model 1		Model 2	
		OR (95% CI)	*P*	OR (95% CI)	*P*	OR (95% CI)	*P*
EFR (kcal/g)	–	1.003 (1.001, 1.005)	0.006^**^	1.004 (1.002, 1.007)	<0.001^***^	1.005 (1.002, 1.008)	<0.001^***^
Group by quartile of EFR	Q1	1.00 (ref.)	–	1.00 (ref.)	–	1.00 (Ref.)	–
	Q2	1.068 (0.615, 1.854)	0.82	1.199 (0.649, 2.213)	0.56	1.217 (0.650, 2.279)	0.54
	Q3	1.359 (0.789, 2.343)	0.27	1.605 (0.878, 2.934)	0.12	1.668 (0.904, 3.079)	0.10
	Q4	1.983 (1.158, 3.396)	0.013*	3.091 (1.656, 5.770)^***^	<0.001^***^	3.546 (1.836, 6.848)	<0.001^***^
Group by quartile of energy (kcal/d)	Q1	1.00 (ref.)	–	1.00 (ref.)	–	1.00 (ref.)	–
	Q2	1.577 (0.925, 2.689)	0.09	1.370 (0.758, 2.476)	0.30	1.621 (0.835, 3.147)	0.15
	Q3	0.925 (0.535, 1.599)	0.78	0.865 (0.472, 1.584)	0.64	1.148 (0.569, 2.314)	0.70
	Q4	1.317 (0.770, 2.253)	0.31	1.388 (0.765, 2.518)	0.28	2.382 (0.991, 5.723)	0.05
Group by quartile of DF (g/d)	Q4	1.00 (ref.)	–	1.00 (ref.)	–	1.00 (ref.)	–
	Q1	1.636 (0.945, 2.834)	0.08	2.036 (1.104, 3.754)	0.023*	2.575 (1.250, 5.305)	0.010*
	Q2	1.860 (1.069, 3.236)	0.028*	2.122 (1.153, 3.908)	0.016*	2.477 (1.269, 4.837)	0.008^**^
	Q3	1.177 (0.672, 2.061)	0.57	1.278 (0.692, 2.361)	0.43	1.346 (0.708, 2.560)	0.36
Covariates	Age (y)	–	–	0.959 (0.939, 0.981)	<0.001^***^	0.961 (0.939, 0.984)	0.001^**^
	Retired	–	–	0.454 (0.262, 0.787)	0.005^**^	0.481 (0.272, 0.852)	0.012*
	High school and above	–	–	1.800 (1.107, 2.927)	0.018*	–	–
	Smoking	–	–	1.801 (1.097, 2.958)	0.020*	1.686 (1.017, 2.796)	0.043*
	Alcohol drinking	–	–	2.087 (1.265, 3.444)	0.004^**^	2.050 (1.230, 3.417)	0.006^**^
	Antihypertensive drugs	–	–	–	–	0.500 (0.290, 0.860)	0.012*

DBP, diastolic blood pressure; EFR, energy to fiber ratio; OR, odds ratios; CI, confidence intervals; Q, quintile; DF, dietary fiber.

^***^p < 0.001;

^**^p < 0.01;

*p < 0.05. Crude: did not adjust anything. Model 1: adjusted for age, sex, occupational status, education degree, smoking, alcohol drinking. Model 2: adjusted for variables in model 1 + body mass index (BMI), duration of hypertension (HTN), taking antihypertensive drugs, protein intake, cholesterol intake.

## Discussion

To the best of our knowledge, this is the first study designed to evaluate the association between EFR and BP control among hypertensive patients. Consistent with our study hypothesis, the present study showed that a higher EFR was a stronger risk factor for BP control among Chinese patients with essential hypertension. Hypertensive patients with a high dietary EFR were at higher risk of poor BP control than those with a low dietary EFR. A higher dietary EFR may reflect higher consumption of calorie-dense foods and lower intake of DF-rich foods such as whole grain, fruits, and vegetables, representing poorer diet quality, consistent with the characteristics of Western dietary patterns (41). In addition, a higher proportion of participants in the present study were male (62.8%). This imbalance may reflect sex differences in hypertension prevalence, health-seeking behavior, and participation willingness in community- and hospital-based investigations in the study region. However, due to the observational design and the sample distribution in the present study, sex-stratified analyses were not performed. Therefore, the potential influence of sex on the association between dietary EFR and blood pressure control cannot be fully excluded. Future studies with larger and more balanced populations are warranted to further explore possible sex-specific differences.

In recent years, China has experienced a shift from traditional to Western dietary patterns, with a decrease in the consumption of grains and vegetables and an increase in the consumption of animal foods, saturated fat, sugar-sweetened beverages (42, 43). These dietary changes coupled with sedentary lifestyles which result in a series of problems such as a positive energy balance and an imbalanced dietary structure (42, 44). In our study, the mean values of energy, DF, and EFR of total participants were 1998.2 kcal/d, 11.3 g/d, and 189.0 kcal/g, respectively. Energy and DF intake are similar to the findings of the 2015–2017 China Health and Nutrition Survey (45), which showed that the average daily energy and DF intake of Chinese residents were 2007.4 kcal and 10.4 g. A total of 83 participants (18.2%) exceeded the recommended energy intake. However, the daily DF intake was far below the recommendation of the 2013 Chinese Dietary Guidelines (25–30 g/d) (39) and only six participants (1.3%) met this recommendation.

The EFR in this study was significantly higher than the found of Guligowska et al.’s (46), conducted among older adults over 60 years of age from Poland, which showed that the EFR was 122 in the depressed group and 89.78 in the normal group. This difference may be attributable to differences in countries and populations. Studies showed that the level of DF intake was higher in European regions including Poland, than in East Asia (5); the elderly have low requirements of energy due to decreased basal metabolism and reduced physical activity (47). In this study, participants were recruited in China (East Asia) thus had a lower DF intake and they were with a mean age of 51.3 ± 12.7 years, thus having a higher energy intake. Only 36 participants (7.9%) met the European Heart Network’s recommendation (108kcal/g) (40), suggesting that the participants in our study had a poor-quality diet. Notably, inconsistent with our expectations that the intake of energy and DF in each group (Table 2) showed that DF intake gradually decreased as EFR increased in the four groups of participants, there was no statistically significant difference in energy intake between the groups. This could be explained by the fact that a dramatic decline in physical activity levels accompanied by rapid urbanization in recent years, the total energy intake of Chinese residents has greatly reduced in recent years (17). We can also find that fewer participants had high energy intake, thus no difference in energy intake was reflected between the four groups. DF is mainly found in foods such as fruits, vegetables, whole grains, and legumes, which are considered as high quality plant-based foods (48). However, the status of almost same energy intake means that the intake of plant-based foods gradually decreased from Q1 to Q4 groups, with participants in the Q4 group consuming a diet dominated by animal-based foods and the Q1 group by plant-based foods (especially high quality plant-based foods).

Elevated SBP is a leading risk factor contributing to the global burden of disease (49). In the Felodipine Event Reduction randomized trial, lowering SBP to a mean of 138 vs. 142 mmHg was accompanied by 27% reductions in fatal and non-fatal stroke and 25%–35% reductions in cardiovascular outcomes (50).

Accumulating evidence suggests that DF can improve BP control (13) and other cardiovascular risk factors (6). On the other hand, several randomized (51, 52) or non-randomized trials (53, 54) have demonstrated the effectiveness of energy restriction diets (ERD) in reducing BP, although only a few studies have included hypertension (>140/90 mmHg) as inclusion criterion. However, fewer studies have explored whether the effect of ERD combined with DF supplementation on BP control is greater than those of ERD or DF alone among hypertensive patients. Saltzman et al. (31) found that the ERD plus supplementation of DF (oats) resulted in greater improvements in SBP and lipid profiles over 6 weeks compared to ERD alone among healthy population. Similarly, another randomized controlled trial of overweight participants conducted by Benítez-Páez et al. showed that the ERD combined with DF (inulin+resistant maltodextrin) supplementation led to a 10-fold higher reduction in BP compared to ERD alone (29). These studies implied that the additive effects of combining ERD with DF on BP might be greater than the individual effects. In this study, a higher dietary EFR was at higher risk of poor SBP control. Compared with that of the lowest EFRs in the Q1 group (EFR ≤ 149.5), the risk of poorly controlled SBP in Q3 and Q4 groups with EFRs of 189.0 to <246.4 and ≥246.4 significantly increased by 2.112 and 7.233 times, respectively. Despite no statistically significant difference in energy intake between the Q1–Q4 groups, the correlations between EFR and SBP control were still stronger than DF (OR_*Q1vs.Q4*_, 5.279; 95% CI, 2.523, 11.049) or energy intake (OR_*Q4vs.Q1*_, 1.744; 95% CI, 0.633, 4.805) alone. This implies that the animal-based dietary pattern (Q4) may be detrimental to BP control in hypertensive patients compared with the plant-based dietary pattern (Q1), which was consistent with other studies (55, 56). From a clinical perspective, the magnitude of these associations is noteworthy. For instance, individuals in the highest EFR quartile exhibited more than a sevenfold increased likelihood of poor systolic blood pressure control compared with those in the lowest quartile, indicating that dietary imbalance captured by EFR may represent a clinically meaningful and modifiable factor associated with blood pressure control. These findings suggest that EFR could serve as a simple and practical screening indicator in routine clinical settings to identify patients at higher risk of inadequate blood pressure control. Unlike complex dietary indices, EFR can be readily estimated using basic dietary information, making it feasible for use in primary care or community-based management. Clinicians and healthcare providers may consider incorporating EFR into dietary counseling by encouraging increased intake of fiber-rich foods such as whole grains, fruits, and vegetables, while moderating excessive caloric intake from energy-dense foods. Such targeted dietary modifications may contribute to improved blood pressure control and overall cardiovascular risk reduction.

In the last decade, plant-based diets have been promoted for their benefits in the management of chronic non-communicable diseases such as obesity and cardiovascular diseases (57). Especially some high-quality plant-based foods such as whole grains, fruits, vegetables, nuts, and legumes which are rich in DF have been found to be beneficial for cardiovascular health and are associated with a reduced frequency of cardiovascular events. However, a purely plant-based diet may be difficult or impossible to achieve long-term compliance and cannot provide an adequate supply of some nutrients (e.g., Vitamin B12, riboflavin, calcium, and iron). In this study, hypertensive patients in the Q1 group with consumption more DF-rich plant foods showed better BP control. These populations were more likely to follow the traditional southern Chinese dietary pattern, which is characterized by a higher consumption of fruits, vegetables, grains with a small amount of red meat (58), which was significantly associated with a lower prevalence of hypertension (59). Gibbs et al. also showed plant-based diets with limited animal products (e.g., DASH, the lacto-ovo vegetarian, and the Nordic diet) lower both SBP and DBP (56).

Although the 2017 American Heart Association/American Heart Association guidelines for the management of hypertension exclude DBP from cardiovascular risk (60), a prospective study of 1.3 million people revealed that DBP also independently predicted adverse cardiovascular outcomes (61), thus DBP should not be overlooked as well. In this study, a higher EFR was also independently associated with a higher rate of poorly controlled DBP. Compared with that of the lowest dietary EFR (≤149.5) in the Q1 group, the risk of poorly controlled DBP in the Q4 group (EFRs ≥ 246.4) increased by 3.546 times. Similarly, the correlations between EFR and DBP control were still stronger than DF (OR_*Q1 vs. Q4*_, 2.575; 95% CI, 1.250, 5.305) or energy intake (OR_*Q4 vs. Q1*_, 2.382; 95% CI, 0.991, 5.723) alone.

1. The pathophysiology of hypertension is highly attributable to vascular endothelial cell dysfunction (37). DF enriched in high-quality plant-based foods can be fermented by gut bacteria to increase the production of short-chain fatty acids (SCFAs) (62, 63). SCFAs can exert an anti-inflammatory role by reducing the secretion of the inflammatory factors and improve insulin resistance (64–66), which can restore endothelial-dependent vasodilation (9, 10), thereby contributing to the improvement of hypertension (11). Moreover, SCFAs regulate the renin-angiotensin-aldosterone system, which in turn lower BP (67). High-quality plant-based foods also contain more vegetable protein, antioxidant nutrients (e.g., polyphenols, vitamin C and E), and micronutrients (potassium, magnesium) that are linked to cardiovascular benefits (57). On the other hand, energy restriction produces the reduction of adipocytes which is beneficial to the reduction of inflammation and oxidative stress levels and improves endothelial NO bioavailability and function, leading to an improvement in BP (68). Therefore, although there was no statistically significant difference in energy intake between groups, relatively subtle but complex interactions between DF and energy intake result in changes in BP, that is, EFR can better predict the impact of dietary quality on BP control in hypertensive people. This finding helps to provide nutritional guidance to hypertensive patients and reduce the burden of cardiovascular disease.
2. Beyond the established role of dietary fiber-derived SCFAs, the relationship between EFR and blood pressure control likely involves multiple interacting biological pathways. First, low dietary fiber intake is associated with reduced gut microbiota diversity and decreased production of beneficial metabolites such as butyrate, which play a critical role in maintaining vascular homeostasis and regulating inflammatory responses (62). Experimental evidence indicates that SCFAs can modulate immune cell activity, suppress pro-inflammatory cytokine production, and improve endothelial function, thereby contributing to blood pressure reduction (64). Second, excessive energy intake—particularly from energy-dense, low-fiber foods—promotes adiposity, systemic inflammation, and oxidative stress, which impair endothelial nitric oxide (NO) bioavailability and vascular function. These mechanisms are closely linked to increased vascular stiffness and elevated peripheral resistance, both of which contribute to poor blood pressure control. Third, EFR may reflect broader dietary patterns characterized by insufficient intake of potassium, magnesium, and antioxidant nutrients, which are essential for vascular regulation and cardiovascular health (57). Diets with high EFR are typically associated with lower consumption of fruits, vegetables, and whole grains, and higher intake of processed and energy-dense foods, thereby further exacerbating cardiometabolic risk. Importantly, EFR captures the combined and potentially synergistic effects of excessive energy intake and insufficient fiber intake, rather than considering these components in isolation. This integrative property may explain why EFR demonstrated a stronger association with blood pressure control compared with energy or dietary fiber alone in our study. Together, these findings suggest that EFR reflects not only nutrient imbalance but also a broader disruption of metabolic and vascular regulatory pathways.
3. Some limitations should not be overlooked in this study. Firstly, dietary intake was assessed using two non-consecutive 24-h dietary recalls, which may be subject to recall bias and may not fully capture habitual dietary intake compared with longer assessment methods such as multi-day dietary records. This limitation may introduce measurement variability and potentially attenuate the observed associations between EFR and blood pressure control. Secondly, due to the cross-sectional design, causal relationships between EFR and blood pressure control cannot be established, and reverse causality cannot be excluded, as individuals with better blood pressure control may adopt healthier dietary patterns. Thirdly, participants were recruited using a convenience sampling approach from both hospital and community settings in Suzhou, which may introduce selection bias and limit the generalizability of the findings. Individuals included in this study may differ systematically from the broader hypertensive population in terms of health awareness, healthcare access, and lifestyle behaviors, which could influence both dietary patterns and blood pressure management, thereby potentially leading to biased estimates of the association between EFR and blood pressure control. In addition, although dietary sodium intake was included as a covariate in the multivariable models, other potential confounders such as psychological stress and dietary supplement use were not assessed due to data limitations. The absence of these variables may introduce residual confounding, which could bias the estimated associations between EFR and blood pressure control.
4. Future research is warranted to further elucidate the role of dietary energy-to-fiber ratio in blood pressure regulation. First, longitudinal cohort studies are needed to establish temporal relationships and clarify potential causal links between EFR and blood pressure control. Second, randomized controlled trials should be conducted to determine whether interventions aimed at reducing EFR—such as increasing dietary fiber intake or optimizing overall dietary patterns—can lead to clinically meaningful improvements in blood pressure outcomes. In addition, future studies may explore the interaction between EFR and other dietary quality indices or nutrient ratios, as well as its relationship with metabolic and inflammatory biomarkers, to better understand its underlying mechanisms and predictive value.

## Conclusion

A higher dietary EFR was significantly associated with poor blood pressure control among Chinese patients with essential hypertension. These findings suggest that EFR may serve as a simple, practical, and clinically accessible indicator for evaluating dietary quality in hypertensive populations. From a clinical perspective, incorporating EFR into routine dietary assessment may help identify individuals at higher risk of inadequate blood pressure control and support more targeted nutritional counseling. Interventions aimed at reducing EFR, such as increasing the intake of fiber-rich foods while moderating excessive energy intake from calorie-dense foods, may contribute to improved blood pressure management. From a public health perspective, promoting dietary patterns characterized by lower EFR may represent a feasible strategy to reduce the burden of hypertension and related cardiovascular diseases.

## Data Availability

The raw data supporting the conclusions of this article will be made available by the authors, without undue reservation.

## Footnotes

1
https://cran.r-project.org/

## Supplementary material

Supplementary Figure 1 The directed acyclic graph (DAG) for the selection of covariates and confounders adjustment.
