# Supplementary figures and images for: The higher dietary energy-to-fiber ratio is associated with an increased risk of poor blood pressure control among patients with essential hypertension: a cross-sectional study
Source: Front Nutr. 2026 May 5;13:1763181. doi: 10.3389/fnut.2026.1763181 (PMC13183529; doi:10.3389/fnut.2026.1763181)

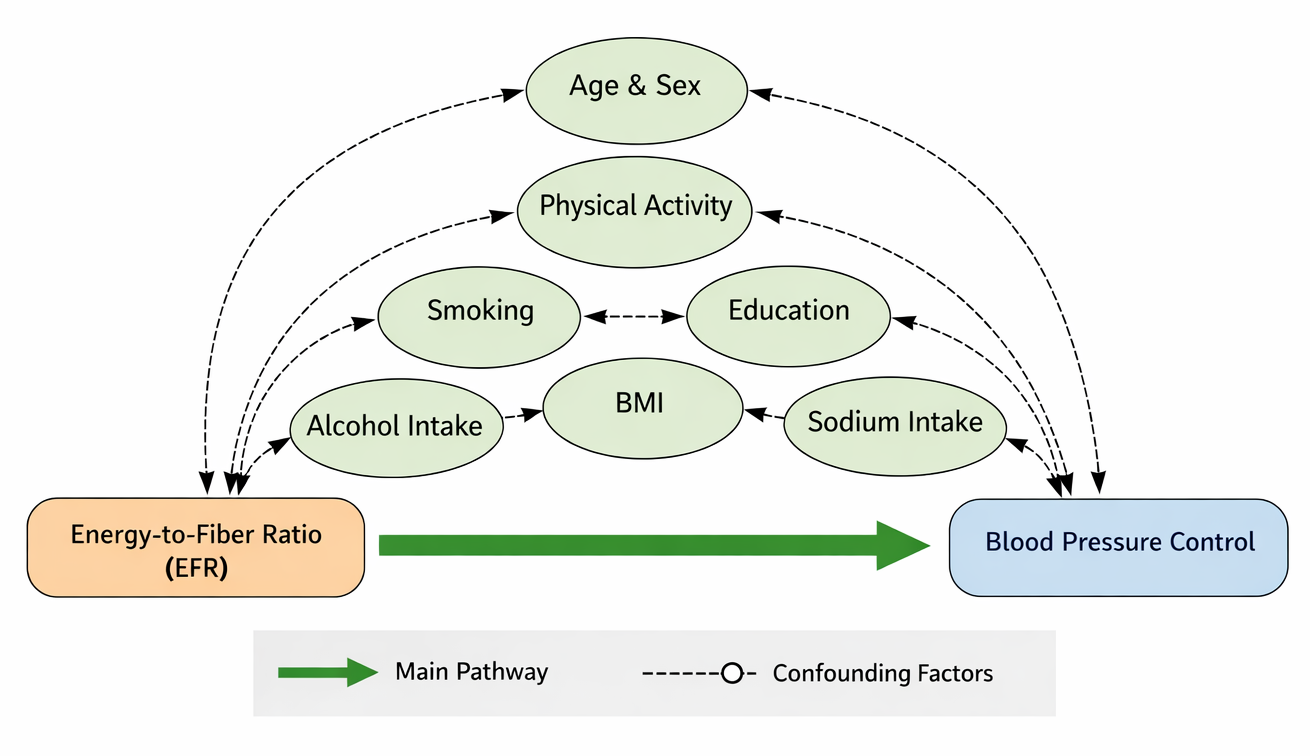

Supplement: Supplementary Figure 1 — The directed acyclic graph (DAG) for the selection of covariates and confounders adjustment. [file Image_1.tif]
